# Isothermal drying characteristics and kinetics of human faecal sludges

**DOI:** 10.12688/gatesopenres.13137.2

**Published:** 2021-04-21

**Authors:** Tosin Somorin, Samuel Getahun, Santiago Septien, Ian Mabbet, Athanasios Kolios, Chris Buckley

**Affiliations:** 1 Chemical and Process Engineering, University of Strathclyde, Glasgow, G1 1XQ, UK; 2 Pollution Research Group, University of Kwazulu-Natal, Durban, 4041, South Africa; 3 Department of Chemistry, Swansea University, Swansea, UK; 4 Naval Architecture, Ocean & Marine Engineering, University of Strathclyde, Glasgow, G1 1XQ, UK

**Keywords:** Thermogravimetric Study, Biomass Conversion, Sanitation Intervention, Kinetic Behaviour, Sludge Treatment

## Abstract

**Background:** Drying is an important step for the thermochemical conversion of solid fuels, but it is energy-intensive for treating highly moist materials.

**Methods:** To inform the thermal treatment of faecal sludge (FS), this study investigated the drying characteristics and kinetics of various faecal wastes using thermogravimetric analysis and isothermal heating conditions.

**Results:** The findings show that FS from the anaerobic baffled reactor (ABR) and ventilated improved pit latrines (VIP) exhibit similar drying characteristics, with maximum drying rates at 0.04 mg/min during a constant rate period that is followed by a distinct falling rate period. On the contrary, fresh human faeces (HF) and FS from urine diversion dry toilets (UDDT) exhibited a falling rate period regime with no prior or intermittent constant rate periods. The absence of a constant rate period in these samples suggested limited amounts of unbound water that can be removed by dewatering and vice versa for VIP and ABR faecal sludges. The activation energies and effective moisture diffusivity for the sludges varied from 28 to 36 kJ/mol and 1.7·10 ^-7^ to 10·10 ^-7^ m ^2^/s at 55°C and sludge thickness of 3mm. The Page model was consistent in modelling the different sludges across all temperatures.

**Conclusions:** These results presented in this study can inform the design and development of innovative drying methods for FS treatment.

## Introduction

More than one-third of the world’s population are without access to modern sanitation, a situation that disproportionately affects low-income countries, particularly rural dwellers ^1^. To eradicate poor sanitation, on-site sanitation facilities are being developed in many parts of the world. This includes the development of i) ecological toilets e.g. ventilated improved pit (VIP) latrines, composting toilets, and urine-diverting dry toilets to safely collect and convert human waste to an environmentally-friendly form (e.g. compost) and for recovery of useful products such as fuel and energy ^2^, and ii) advanced waste-to-energy technologies to convert human waste to fuel, heat and/or electricity, without putting undue pressure on natural resources ^3, 4^. For safe handling, transportation and environmental quality of faecal waste streams, processes such as dewatering, drying and pasteurisation are recommended ^5^.

Thermal drying is of specific interest because it can reduce waste volumes and improve the longevity and quality of end-products ^6^. The integration of heat can eliminate pathogens and odour with potential health and environmental gains, but the scale of benefits in faecal sludge management will depend on material characteristics and process conditions ^7, 8^. To eliminate pathogens in waste streams, sufficiently high temperature and residence time need to be reached ^9^, which can come at a cost when large volumes of waste are treated. If the dryer is not appropriately designed, drying could reduce the energy quality of the feedstock. Material characteristics can change, causing properties such as stickiness, viscosity and shrinkage to initiate wear and tear of moving parts of internal combustion engines. Auto-ignition can occur, which might lead to an event of a fire ^10^. Thus, an appropriate drying method is important for the safe removal of moisture. In this regard, thermogravimetric techniques can provide insights into the drying characteristics of faecal sludges and kinetic processes governing internal mass transfer.

Thermogravimetric analysis (TGA) enables material characterisation because it measures the change in mass of a material with respect to time or temperature, as the material is subjected to controlled temperature and heating changes ^11, 12^. Based on TGA techniques, drying rates are shown to vary from one sludge to another, depending on sample composition, origin, treatment method and retention time ^13, 14^. Here, intrinsic material properties such as porosity, density, water-solubility, thermal conductivity and viscosity affect drying rates, alongside with environmental factors (e.g. air velocity, temperature and humidity). The rate of loss of moisture differs in treated and untreated sludges and the volume of shrinkage, energy requirement and drying kinetics have changed with the type of additive used ^15, 16^. Studies by Zhang *et al.* ^17^ showed that municipal sewage sludge (MSS) has multiple drying profiles with characteristic apparent activation energies and effective moisture diffusivities. In the study conducted by Qian *et al.* ^18^, multiple falling rates were not reported but drying mechanisms follow a shrinkage core model with drying temperature and sample mass influencing drying kinetics. The multiple drying profiles observed by Reyes *et al.* ^19^ for MSS in a drying tunnel with parallel airflow at various temperatures and air velocities exhibited a relatively long constant rate period. Drying curves were modelled using Fick’s second law equation and quasi-stationary methods. Among the nine different mathematical models employed by Zhang *et al.* ^16^, the Midilli model outperformed others, with respect to the prediction of the moisture content evolution during drying in the tested sludge, and in comparison, to experimental data. Thermogravimetric methods and kinetic models are, thus, proven tools for understanding and modelling the drying behaviour of sludge.

Several studies that have evaluated drying behaviour of sludge materials have focused on materials such as sewage sludge ^20^, pulp wastes ^21^ using different dryer configurations, contact methods (direct or indirect) and operating conditions to identify and optimise process efficiency ^21, 22^ but limited information exists on FS, which differ in material composition ^23^. A few studies that have attempted to understand FS drying have given focus to the development of physical processes ^24– 26^. To accommodate the unusual proprieties of FS and minimise capital and operating costs in off-grid systems, low-cost dewatering approaches such as drying beds, geobags, Imhoff tanks, membrane envelopes, were often cited. In this respect, Cofie *et al.* ^24^ described drying beds as an effective method for dewatering and removing helminth eggs from FS but results varied and depended on the quality of the filtering medium, degree of stabilisation, loading rates, bed height and on external conditions (e.g. rainfall and ambient temperature). Long residence times, which are in the order of 1 – 8 weeks, high land space requirement and the need to treat the resulting effluent (percolate) further limited their application. Seck *et al.* [25a] showed that drying rates can be improved in drying beds by mixing FS during loading but covering the bed (e.g. using greenhouse structure) provided no significant additional benefits apart from providing rain shield. Other treatment methods involving geobags and Imhoff tanks had reduced land requirements, but pathogen loads were only slightly reduced and post-treatment was needed to sanitise solid and effluent waste streams ^2, 27^. Thermal processes, e.g. “LaDePa” (Latrine Dehydration Pasteurization) infrared dryer and solar dryers, have been proposed and are being developed but further information is needed to improve dryer performance, decrease area footprint and to reduce energy consumption.

This study presents the drying characteristics and kinetics of onsite sanitary wastes under controlled isothermal heating conditions. Temperatures between 55°C and 205°C were considered in this study to model low heating and pre-treatment conditions. FS from different sources were investigated to account for compositional changes. Kinetic parameters associated with drying were determined using mathematical models (Page, Newton, Logarithmic and Henderson). The results presented in this study can inform the design of innovative drying methods for FS treatment. The kinetic data can serve as reliable process model inputs for thermal drying treatment of FS.

## Methods

### Sample preparation & characterisation

On-site sanitary wastes were received from the Pollution Research Group, at the University of KwaZulu-Natal, South Africa. The sludges that were collected during pit emptying of on-site sanitation facilities in the eThekwini municipality (Durban metropolis, South Africa), include: a) anaerobic baffled reactor (ABR) at a decentralized wastewater treatment system (DEWATS); b) VIP latrines and c) urine-diverting dry toilet (UDDT). The DEWATS is a mixture of black water, greywater and human faecal sludges and it receives effluent from neighbouring households and a communal ablution block in Fraser's informal settlement, Durban. Due to the limited size of the inlet of the DEWATS, samples were collected during pit emptying and using a vacuum truck. For representative sampling of the DEWATS, samples were collected from three zones (top, middle and bottom) of the settling tank (first compartment). The VIP samples were collected from a pit in Bester informal settlement, located 25 km north of Durban. Due to inaccessibility of the pit, FS was collected directly from a vacuum truck during pit emptying; however, this process involves water dilution for suction. The FS sample from the UDDT was collected from Kwamashu, 20 km north of Durban, a facility that serves a single household. FS samples from the UDDT were collected manually using spades and forks, with large household waste (clothing, sanitary material, paper, etc.) found in the sludge removed onsite. All samples were screened for materials larger than 5mm (using a 5mm sieve) and then stored at 4°C at the laboratory of the Pollution Research Group. The sampling procedure and samples characteristics is described with more detail in Getahun *et al.* ^28^.

The collection and analysis of FS for this investigation were approved by the Biomedical Research Ethical Committee from the University of KwaZulu-Natal (Ethical Clearance Reference: EXM005/18). The samples were couriered to Cranfield University in sealed plastic bottles to avoid moisture loss, wrapped in zip lock bags and contained in a box with ice blocks between the secondary and outer packaging for continuous preservation of samples at 4°C. This was under the authorization of the Health Department of the Republic of South Africa (export permit: J1/2/4/2). At Cranfield University, fresh human faeces (HF) was collected from a volunteer (single donor) as part of the Nano Membrane Toilet Sampling Collection Campaign. This sample collection process involves: a) campaign for voluntary donation of human faeces at Cranfield University from staff and students, b) preparation and provision of sample collection kits (including cardboard bowl, pair of gloves, black plastic bags (for collection and disposal of gloves), zip ties as well as information and instruction sheet, volunteer consent sheet, pen and labelling paper in designated sampling toilets, c) anonymous donation of sample in a cardboard collection bowl contained in a black plastic bag with zip ties and held in a plastic box, d) collection of samples from designated sampling toilets, e) sample storage at -85°C in a designated freezer. This sampling protocol is approved by the Cranfield Research Ethics Committee Approval (CURES/2310/2017) and consent is agreed in written form by completing a volunteer consent form. Due to the focus on drying, the HF sample was stored at 4°C and brought to room temperature before analysis. To determine the initial moisture content of the samples, 5g of the samples was weighed and dried at 105 ± 5°C in a hot air oven. Table 1 highlights the initial moisture content of the samples as received from the University of KwaZulu-Natal and Cranfield University. Average values of moisture content at the time of analysis as determined using hot air oven and average equilibrium moisture as determined by TGA are also reported.

**Table 1.** Initial moisture content of samples (as received basis).

	ABR	HF	UDDT	VIP
Moisture content measured in South Africa (wt. %) ^28^	88	62 *	70	95
Moisture content measured before TGA tests (wt. %)	88 ± 3	75 ± 1	65 ± 0	95 ± 2
Equilibrium moisture as determined by TGA (wt. %)	88 ± 3	75 ± 2	65 ± 1	96 ± 1

ABR, anaerobic baffled reactor; HF, human faeces; UDDT, urine-diverting dry toilet; VIP, ventilated improved pit. *as determined in Cranfield.

The moisture content was determined on a wet weight basis using Equation 1.

Moisturecontent(wetbasis,%)=wi-wfwi×100Eqn.(1)

where *w _i_* and *w _f_* are initial and final weight of the sample respectively. 

### Thermogravimetric analysis

Under isothermal drying conditions, 40 mg of sample was thinly spread in a cylindrical aluminium crucible to a diameter of ~3 mm and weighed to an accuracy of ± 0.5 mg. Prior to analysis, each of the samples was mixed manually to achieve uniform consistency/sample homogeneity and mixing is a common process in drying operation. Samples were subjected to controlled temperatures of 55, 85, 105, 155, 205°C in a thermogravimetric analyser (Model: *PerkinElmer TGA 8000™*). All experiments were carried out at gas flow rate of 40 mL/min using synthetic air (with 21% pure oxygen and 79% pure nitrogen and <2 vpm moisture) as the carrier gas. The gas flow rate was selected based on method validation following the use of N2 and air, as well as to maintain uniformity of method across projects, that is for follow-on FTIR/NMR and evolved gas analysis (results not included). Prior to isothermal temperature, samples were raised from 30°C to the specified temperature at a rapid heating rate of 100°C/min to minimise drying during the heating-up stage. For repeatability, each test was conducted at least in duplicates.

### Kinetic analysis: isothermal drying

The moisture ratio (MR), which shows the extent to which drying has taken place in the samples at a given time, was determined using Equation 2.

MR=m–memo–meEqn.(2)

where *m _o_*, *m* and *m _e_* are initial moisture, the moisture of the sample at a time (t) and final moisture of the sample, respectively. The *m _e_* represents the point at which the sample weight is constant with time after drying has stopped after reaching the thermodynamic equilibrium. In this study, the sample was considered to be completely dried at the end of the experiment, so *m _e_* was equal to 0. The drying curves were fitted with widely used drying models – see Table 2 using MATLAB R2019a Curve Fitting Toolbox (open source alternatives such as GNU Octave could also be used for this purpose).

**Table 2.** Expressions for modelling MR, drying rate and drying time.

Drying model	Moisture ratio ( *MR*)	Drying rate ( *dMR/dt*)	Drying time ( *t*)
Page	*MR = exp(-kt ^n^)*	*dMR/dt = knt ^n-1^ e ^-kt ^n^^*	*t = (-ln MR/k) ^n-1^)*
Newton	*MR = exp(-kt)*	*dMR/dt = ke ^-kt^*	*t = -ln MR/k*
Logarithmic	*MR = a + b exp(-kt)*	*dMR/dt = kne ^-kt^*	*t = (-ln MR/k)/b*
Henderson	*MR = a exp(-kt)*	*dMR/dt = kne ^-kt^*	*t = (-ln MR/k)/b*

The drying curves were fitted with widely used drying models – see Table 2 using MATLAB R2019a software (Curve Fitting Toolbox). These models are empirical and thus their parameters lack of physical significance. The objective of this study was to identify the empirical model that fits the best the drying kinetics and that can be then used by sanitation practitioners for process design, control and optimization. The effective moisture diffusivity of the samples was determined at different temperatures, using Fick’s second law of diffusion in Equation 2– Equation 6. These equations and derivatives are well-known for describing the internal mass transfer mechanisms, especially during the falling rate period. The equations assume that there is a uniform distribution of moisture, negligible external resistance and shrinkage, and constant diffusivity of moisture across each sample. Note that the effective moisture diffusivity is a value that lumps the contribution of several internal mass transfer phenomena, such as gas and liquid molecular diffusivity, capillary movements, flow due to gradients of pressure, among other phenomena.

$$\frac{\partial MR}{\partial t}=\nabla \left[D_{\text{eff}}\left(\nabla MR\right)\right]\;\text{Eqn}.\;(3)$$

$$MR=\frac{8}{\Pi^{2}}\sum_{n=0}^{\infty }\frac{1}{{\left(2n+1\right)}^{2}}\text{exp}\left[\text{-}\frac{\left(2n+1\right)\;\Pi^{2}\;D_{eff}\;t}{4L^{2}}\right]\;\text{Eqn}.\;(4)$$

where *D _eff_*, *L* and *t* are effective moisture diffusivity (m ^2^/s), the thickness of sample in the crucible (m) and drying time (s), respectively. Equation 3 can be simplified to a straight-line through mathematical manipulations, as displayed in Equation 4. As such, a plot of *ln MR* against *t*, as shown in Equation 5, provides the slope of the line, *k*_0_ ( Equation 6), from which the *D _eff_* was derived.

$$\text{ln}\left(MR\right)=\text{ln}\;\left[\frac{8}{\Pi^{2}}\right]\text{-}\left[\frac{\Pi^{2}\;D_{\text{eff}}}{4L^{2}}t\right]\;\text{Eqn}.\;(5)$$

$$k_{0}=\frac{\Pi^{2}\;D_{eff}}{4L^{2}}\;\text{Eqn}.\;(6)$$

Considering the Arrhenius equation in Equation 7 that describes the temperature dependence of *D _eff_*, the activation energy could be obtained from the plot of *ln (D _eff_)* against *1/T(K)*.

$$D_{\text{eff}}=D_{0}\;\text{exp}\left[\frac{E_{a}}{RT}\right]\;\text{Eqn}.\;(7)$$

where *E _a_* is activation energy of the drying process (kJ/mol), *D _o_* is a pre-exponential factor (m ^2^/s), *T* is drying temperature (K) and *R* is universal gas constant (J/mol K). Due to shrinkage and other structural deformation effects that become dominant at later drying regimes as moisture levels reduce to a minimum, this study has considered Deff values for moisture content >20 wt. excluding warm-up/ramping to set temperature in TG curves.

### Statistical analysis of drying models

The statistical analysis was completed using MATLAB R2019a software (Curve Fitting Toolbox). The analysis involved the fitting of drying models in Table 2 for MR to experimental data and comparison of the results using non-linear regression analysis. Two statistical parameters ( Equation 7– Equation 9): i) coefficient of determination (R2) and ii) root mean square error (RMSE), were used to determine the goodness of fit of the predicted values to the experimental data.

$$R^{2}=1-\left(\frac{\sum_{i=1}^{N}{\left(MR_{pre,i}-MR_{exp,i}\right)}^{2}}{\sum_{i=1}^{N}{\left(MR_{pre}-MR_{exp,i}\right)}^{2}}\right)\;Eqn.\;(8)$$

$$RMSE={\left[\frac{1}{N}\sum_{i=1}^{N}{\left(MR_{pre,i}-MR_{exp,i}\right)}^{2}\right]}^{\frac{1}{2}}\;Eqn.\;(9)$$

where *MR _pre,i_* are the predicted moisture ratios, *MR _exp,i_* are the experimental moisture ratios, *N* is the number of observations. The models with the maximum R ^2^ and minimum RMSE was selected as best fit to forecast the drying behaviour of the sludge.

## Results and discussion

### Isothermal drying behaviour

**Figure 1.**
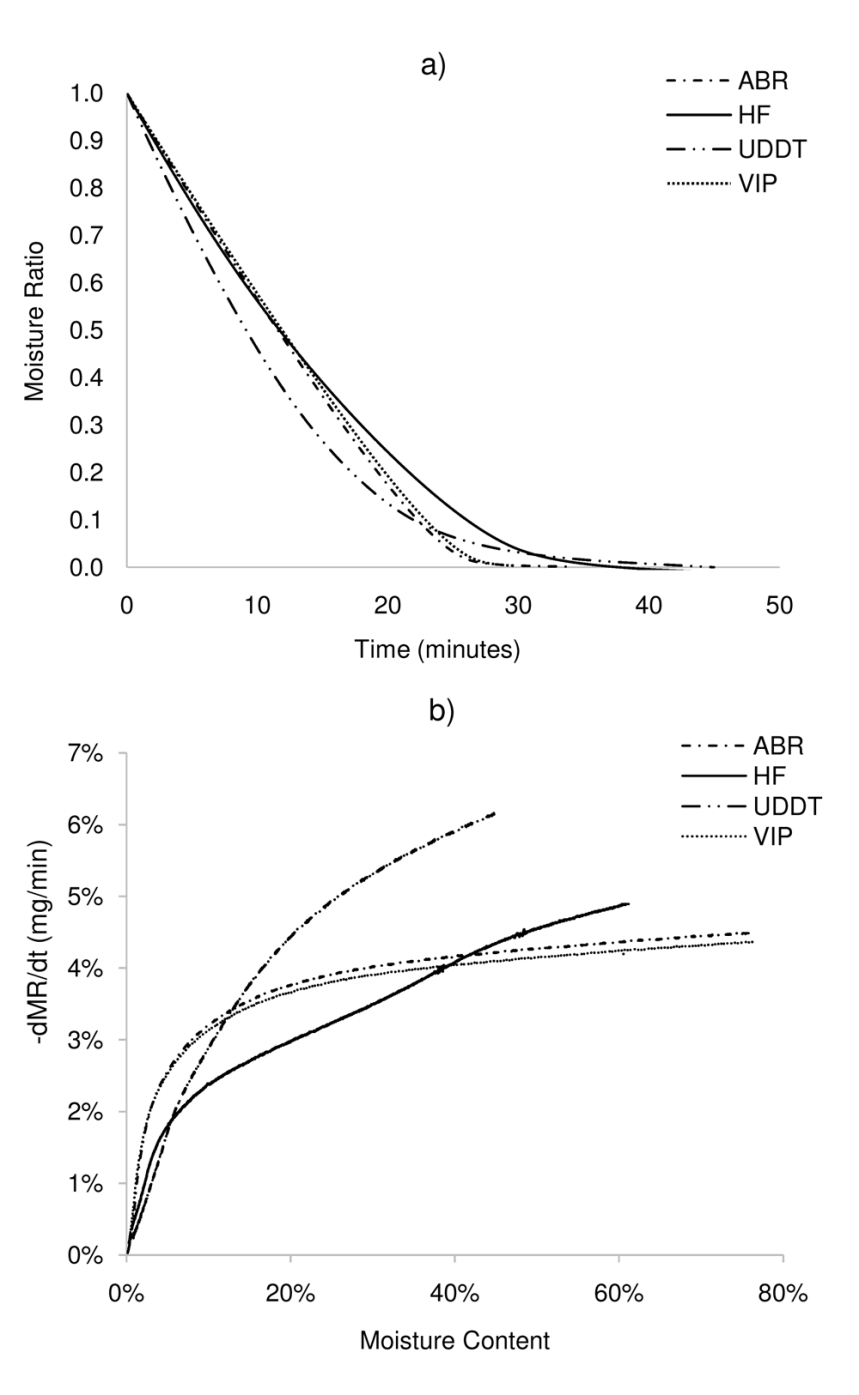
The isothermal drying curves of ABR, HF, UDDT and VIP at 55°C. **a**) moisture ratio vs time, **b**) moisture ratio derivative (drying rate) vs moisture content. ABR, anaerobic baffled reactor; HF, human faeces; UDDT, urine-diverting dry toilet; VIP, ventilated improved pit.

The drying profiles of the ABR, HF, UDDT and VIP at a drying temperature of 55°C are shown in Figure 1 by means of a) MR vs time and b) drying rate vs moisture content (wet basis).

The results in Figure 1a show that MR decreased with time for all the sample types but the drying profiles for some of the samples differed from one another ( Figure 1b). For example, the drying rates for the ABR and VIP samples were relatively constant at ~0.04 mg/min until moisture levels of about 20 wt.% when drying rates started to decrease. However, the drying curves for the HF and UDDT exhibited a falling rate regime with no prior or intermittent constant rate period, with the rate of moisture loss differing across stages. While the ABR sample had a single falling rate period, the HF had multiple falling rates ^29^. These different drying profiles suggest the physical state of water inside the sludge and internal moisture transport processes in the sludges differ from one to another, although the exact mechanisms are not known. Note that, according to the theory, the drying kinetics are mostly controlled by external transfers during the constant rate period, while the falling rate is kinetically controlled by a combination of internal and external transfers (first falling rate period) or only internal transfers (second falling rate period).

Typically in sludges, moisture is said to be present as free, interstitial, surface and/or intracellular water ^30^. These moisture forms exhibit different drying profiles and are affected by the type and strength of chemical bonds in water molecules. According to Erdincler *et al.* ^31^, only the free water and a part of the interstitial water can be removed by conventional dewatering processes, the rest require drying. Free water is considered to have no or loose bonds with particles ^30, 31^; hence, moves freely and can be separated relatively easy by mechanical separation methods ^32^. The interstitial water is said to be bound by active capillary forces within the sludge particles, particularly by microbial flocs that aggregate and form complex links. These can be separated relatively easy by agitation or other mechanical methods such as centrifugation ^10^. The surface water is bound by adhesive forces to particles, with no free movement and includes water that is bound within exopolysaccharides of microbial cells. According to Rose *et al.* ^33^, about 50 wt.% of the moisture in HF are bound in bacterial cells and in complex biofilm matrix – mainly exopolysaccharides. The intracellular water is deemed as bound water, along with surface water, as such, not readily removed by simple solid separation techniques ^34^. These drying concepts suggest that the process by which moisture is being transported and removed from the FS samples are different, although the exact mechanisms for these internal transport processes are not known.

One important material property that is vital for understanding the drying profiles of materials is the critical moisture content ( *X _C_*), which can serve as an indicator of the amount of unbound and bound moisture in the sludge at a low drying temperature. This property indicates the magnitude of the heat and mass transfer resistances in solids ^35^ and changes with material property (e.g. thickness) and factors that affect drying rate (e.g. temperature). In practice, drying will occur at a constant rate if the moisture content is above the critical moisture content and the moisture removed during this period will be considered mainly as unbound. In contrast, below the *X _C_*, the drying rate will exhibit a falling rate regime and the remaining moisture to remove from the sludge will be considered mostly as bound. Indeed, the *X _C_* is an indicator of the amount of unbound and bound moisture in the material. In this study, the *X _C_* for ABR and VIP samples was about 20 wt.%, whereas no *Xc* was observed for the UDDT. For the HF, there was also no prior or intermittent constant rate period, but the multiple falling rates indicate a lower critical moisture content at about 34 wt.%. The high moisture content in VIP and ABR samples and the constant rate period exhibited during drying up to 20 wt.% suggest that unbound or weakly bounded moisture are largely present and part of it can be removed by dewatering. In the case of HF and UDDT, only thermal drying can be applied to remove moisture, because of the likely limited amounts of unbound moisture in the samples. These aspects have implications for the design, development and optimisation of treatment systems. Further work is needed to ascertain the mechanisms by which moisture is held freely in open pores, trapped, locked or bound to organic materials and removed from solids. This can be achieved using advanced imaging techniques and computational methods. Note that at 55°C, drying occurred in a similar way between the VIP and ABR samples, whereas the drying curves were slightly different for the UDDT and HF samples. The UDDT sample exhibited the fastest drying, whereas the HF samples had the lowest drying ( Figure 1).

### Influence of drying temperature

**Figure 2.**
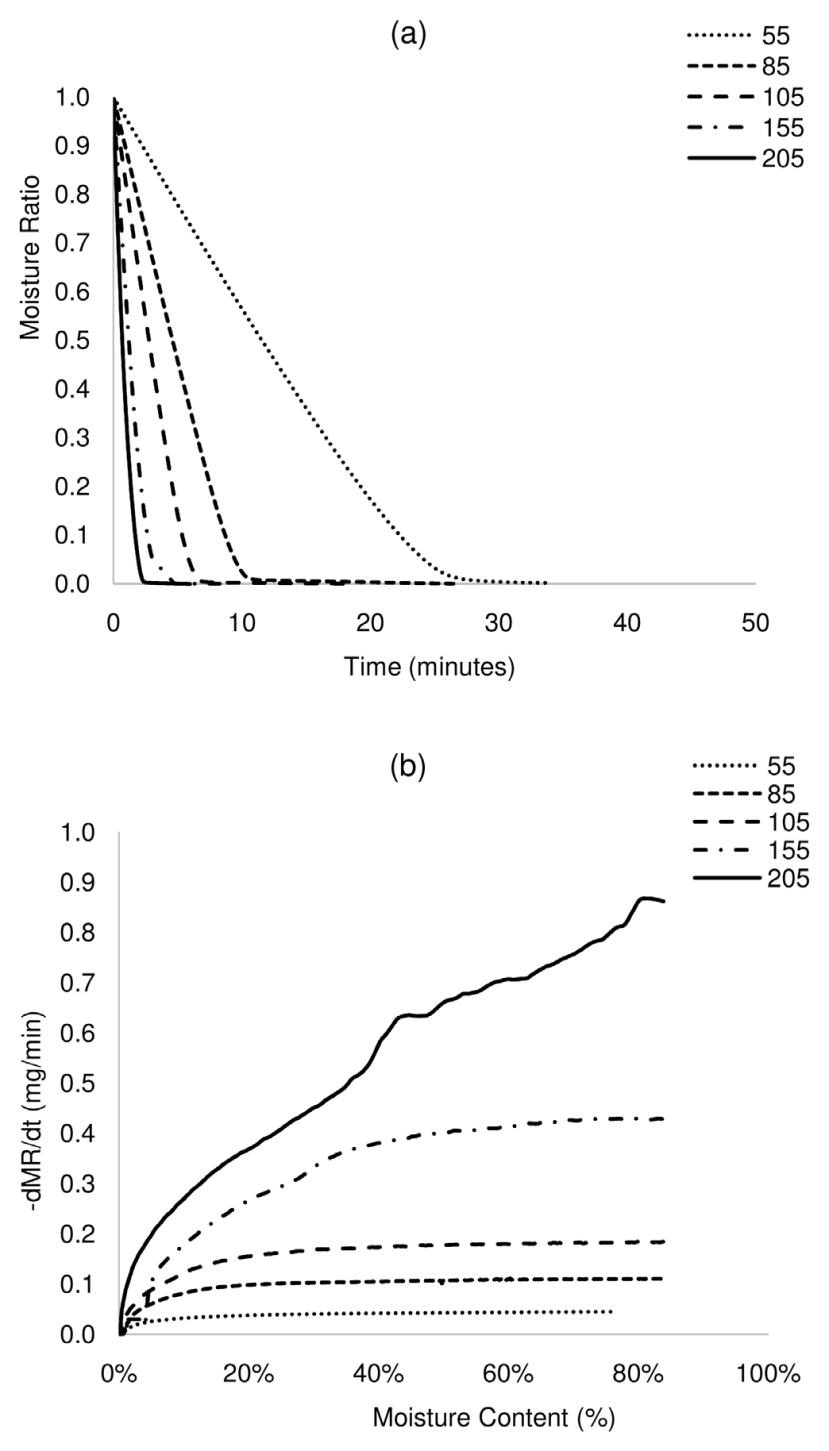
Isothermal drying curves of ABR at 55 – 205°C. **a**) MR versus time and **b**) drying rate versus moisture content. ABR, anaerobic baffled reactor.

The faecal sludges (ABR, HF, UDDT and VIP) were subjected to low-temperature isothermal drying conditions (temperatures: 55 to 205°C). Results are illustrated in Figure 2 – Figure 5 as a plot of a) MR versus time and b) drying rate versus moisture content.

**Figure 3.**
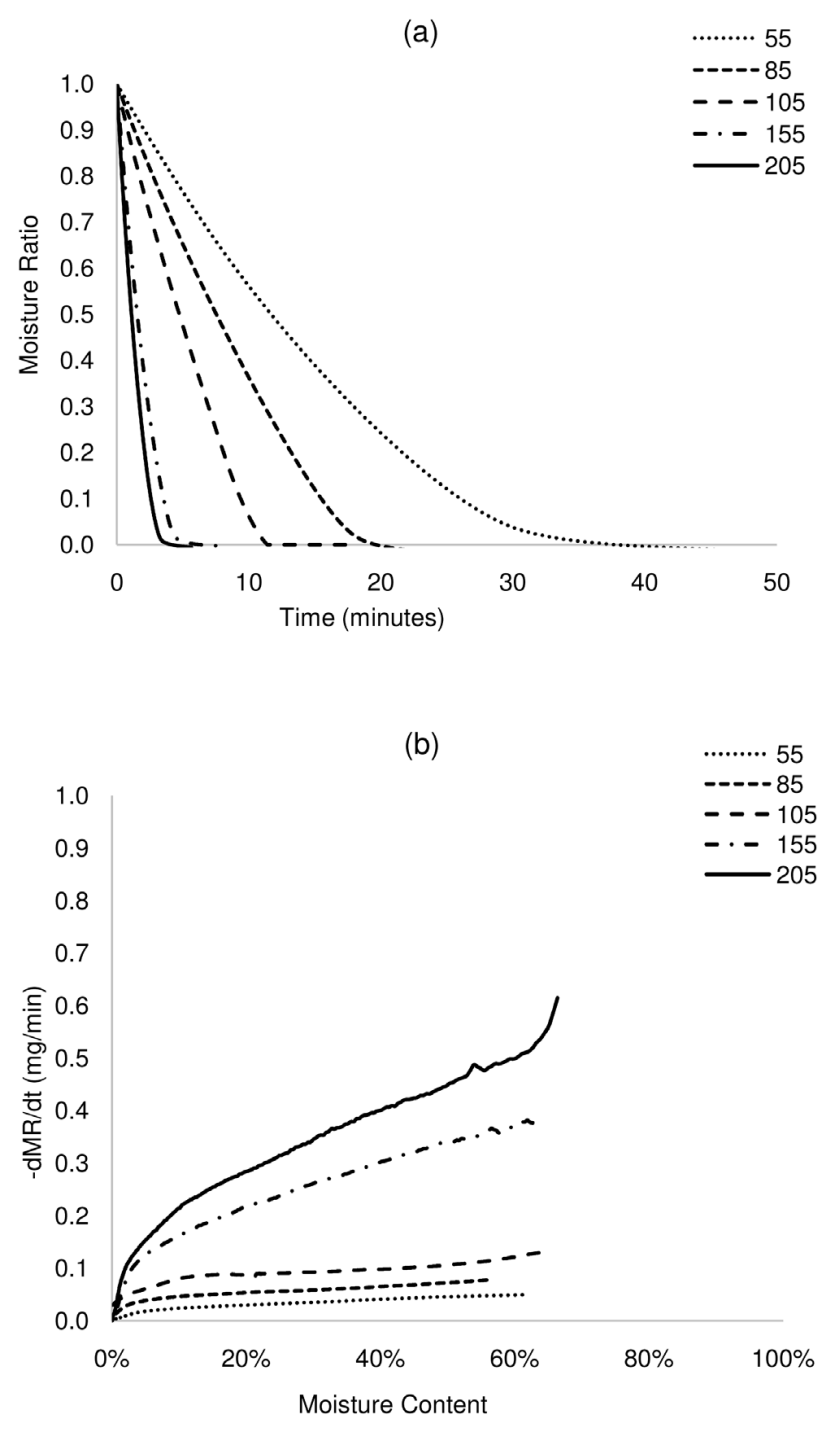
Isothermal drying curves of HF at 55 – 205°C. **a**) MR versus time and **b**) drying rate versus moisture content. HF, human faeces.

**Figure 4.**
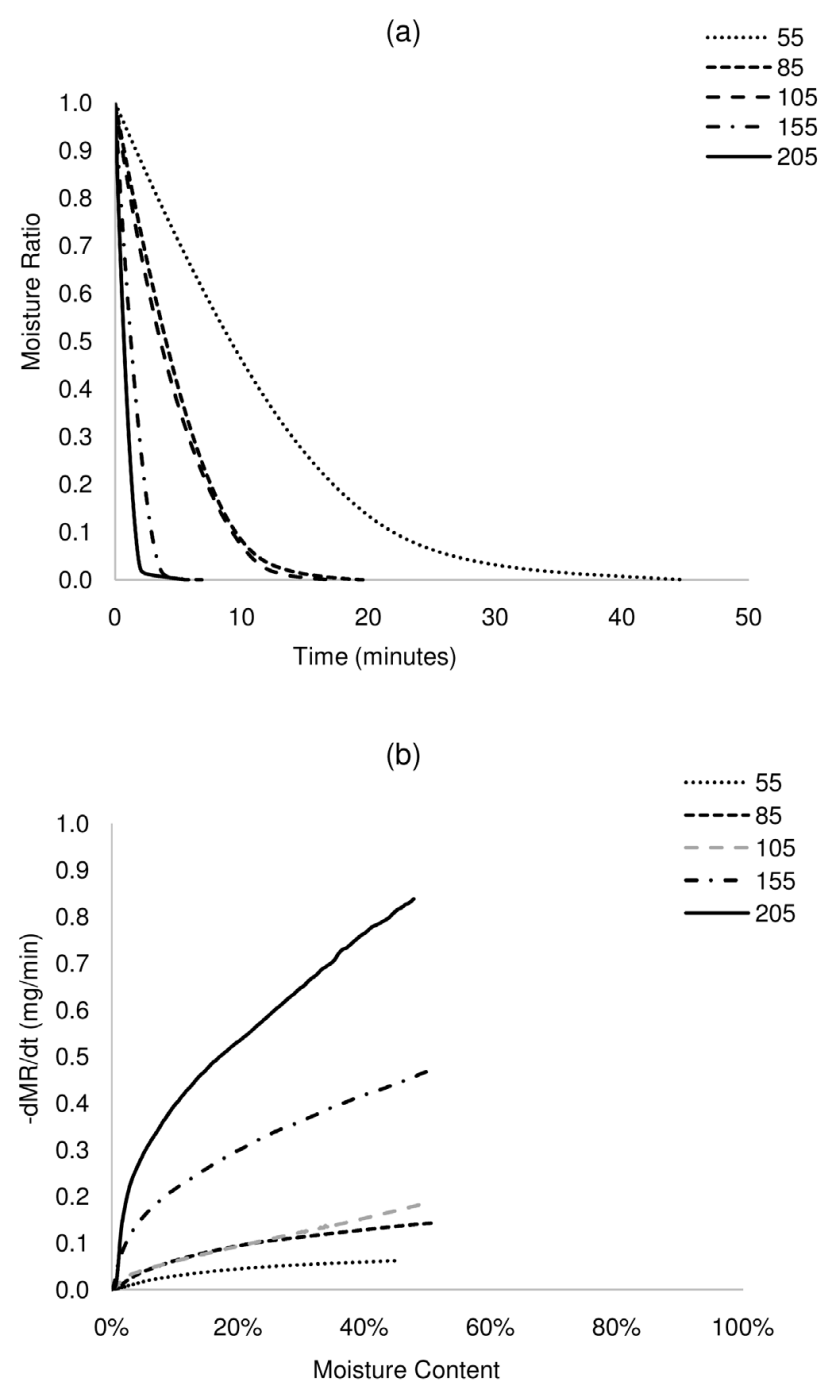
Isothermal drying curves of UDDT at 55 – 205°C. **a**) MR versus time, **b**) drying rate versus moisture content. UDDT, urine-diverting dry toilet.

**Figure 5.**
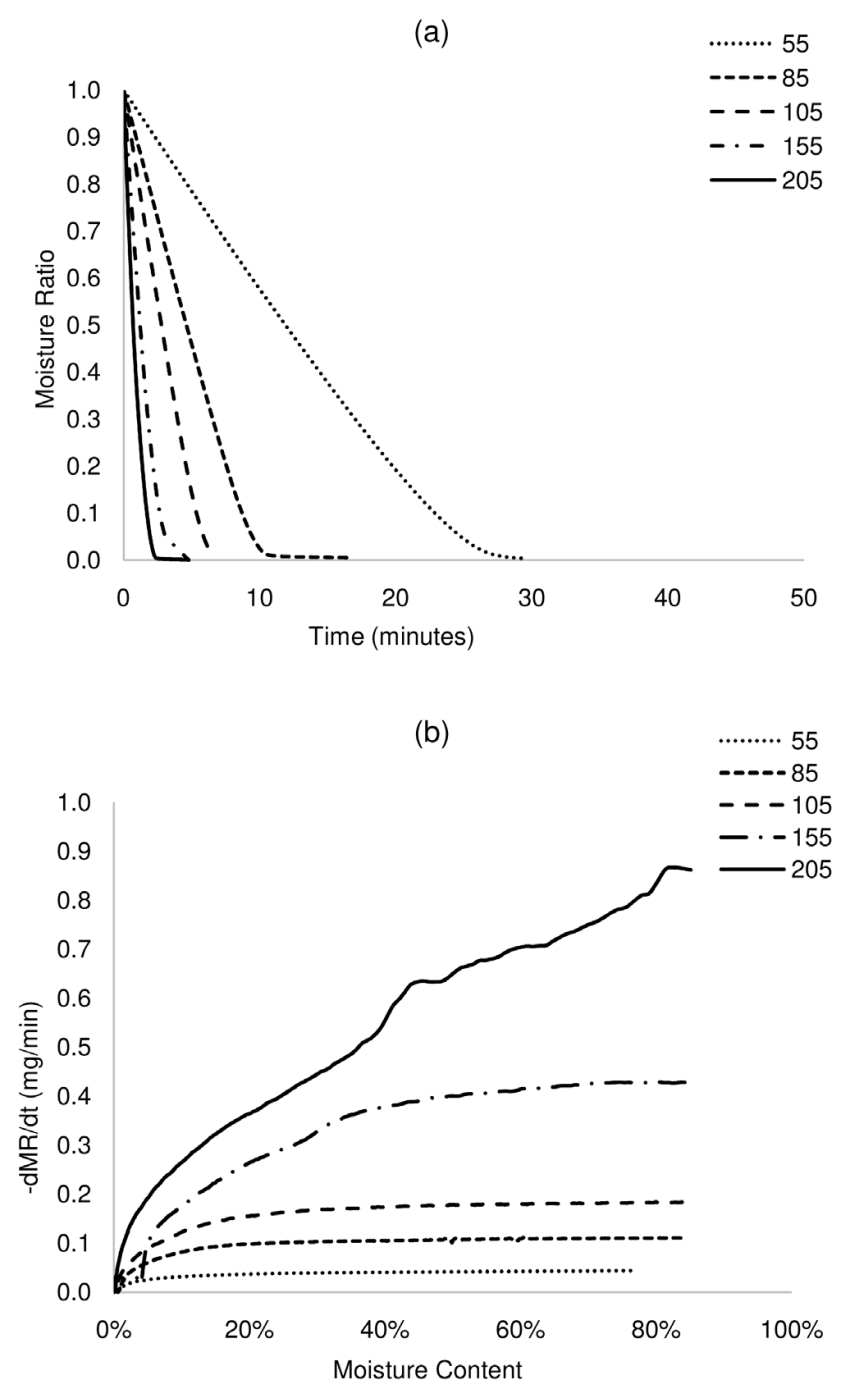
Isothermal drying curves of VIP at 55 – 205°C. **a**) MR versus time, **b**) drying rate versus moisture content. VIP, ventilated improved pit.

The plot of MR against time shows that drying times reduced significantly with an increase in drying temperature ( Figure 2a– Figure 5a). For complete removal of moisture in all the samples, drying times reduced by more than 90% at 205°C, between 64 and 77% at 105°C and up to 62% at 85°C, with respect to drying at 55°C. The drying rates differed across samples, although drying rates increased with increasing temperature. For ABR and VIP samples at a drying temperature of 105°C ( Figure 2b and Figure 5b), the drying rates were relatively constant at 0.18 mg/min until a moisture content of ~20 wt.% (critical moisture content), after which the drying rate started to decrease. A similar pattern was followed at 155°C, but with a higher maximum drying rate (~0.42 mg/min) and shorter constant rate period. At 205°C, drying presented a short constant rate period, and most of the transformation occurred in a falling rate period. Indeed, it can be noted that the constant rate period was shortened as temperature increased. This can be attributed to the effect of temperature on the transport mechanisms. At lower temperature, there is gradual and effective heat transfer into the internal part of the solids, which favours evaporated moisture at the surface of the sludge, maintaining the constant rate period during which the surface of the sludge is completely saturated in moisture. However, at high temperature, transport mechanisms are overtaken by thermal events. Here, the evaporation of the moisture at the sludge surface occurs faster than its replacement with moisture from the core, leading to the decline of the drying rate. In the case of HF and UDDT samples ( Figure 3b and Figure 4b), drying rates also increased and drying times reduced with increasing temperature, but no constant rate period was observed, as commented in the previous section. For all the samples, the falling rate period was the time-consuming step. These results give valuable information for the design and operation of drying systems.

### Kinetic analysis

**Figure 6.**
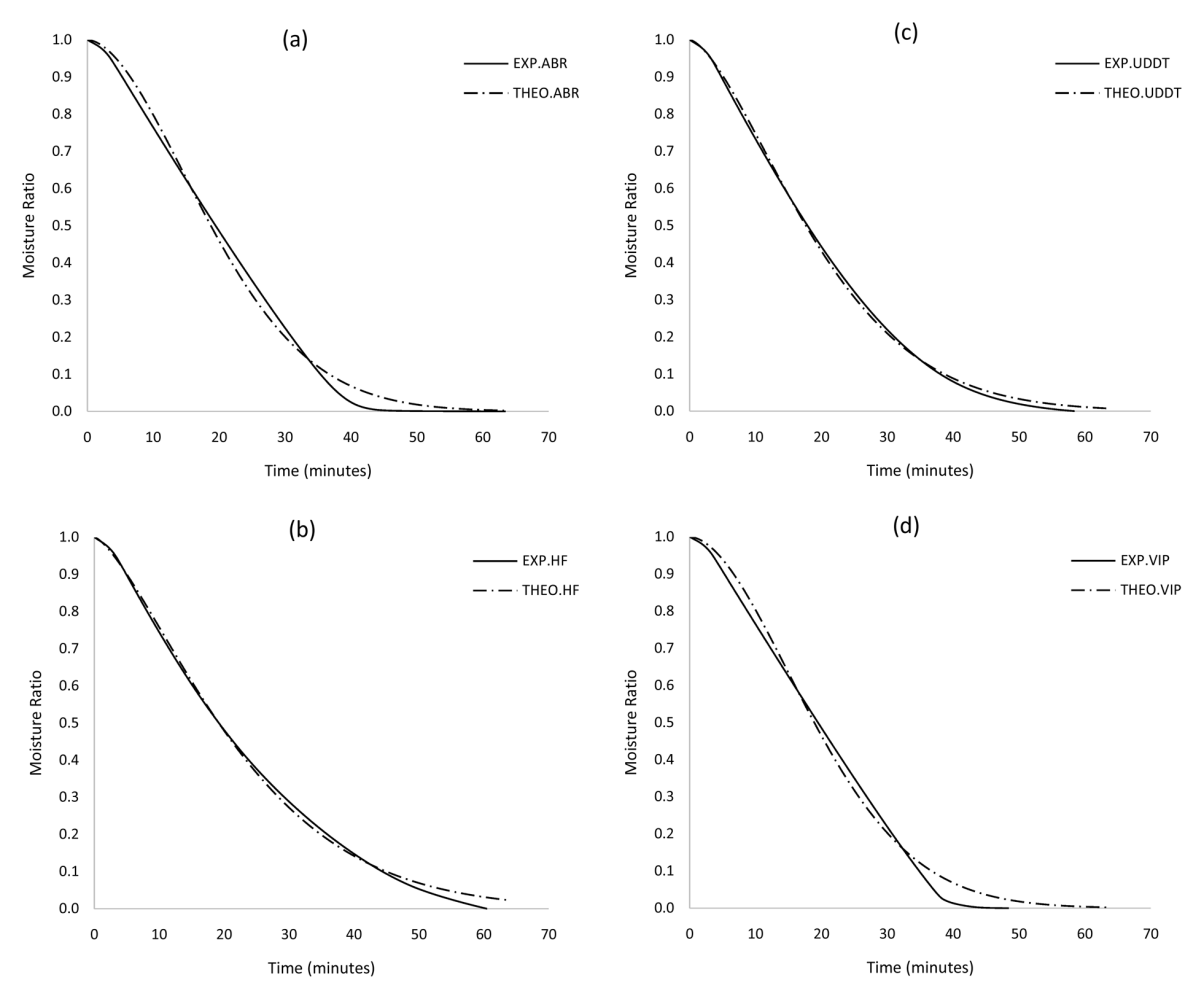
Predicted MR (using Page model) in comparison to experimentally determined values for different faecal sludges at 55oC: **a**) ABR, **b**) HF, **c**) UDDT and **d**) VIP. ABR, anaerobic baffled reactor; HF, human faeces; UDDT, urine-diverting dry toilet; VIP, ventilated improved pit.

The plots of MR versus time were fitted into Page, Newton, Logarithmic and Henderson models ^35^. The selection of the most appropriate model was based on statistical indicators ( *R ^2^* and *RMSE*). The higher the values of *R ^2^* and the lower the values of *RMSE*, the better the measure of fitness of the predicted values to the experimental data and predictive power to model the drying kinetics of the sludges. The results, which are summarised in Table 3 – Table 6, show that the Page model best describes the drying profiles of the various sludges, particularly the UDDT. The experimentally determined and predicted data points for the Page Model are illustrated in Figure 6a – Figure 6d. For the Page model, *R ^2^* values exceeded 0.992 and *RMSE* were at most 0.03. For the other models (Henderson, Netwon and Logarithmic), *R ^2^* values varied between 0.844-0.981, 0.769-0.962 and 0.934-0.998 respectively. The corresponding *RMSE* values for the other models were between 0.04-0.160, 0.04-0.195 and 0.01-0.104. However, it is worth mentioning that the page model slightly overestimates the latter drying regime and these could be due to shrinkage effects that have not been considered. Due to this, we have considered D _eff_ for moisture content >20 wt. excluding warm-up conditions in TG curves.

**Table 3.** Statistical data for isothermal drying of anaerobic baffled reactor (ABR).

Model Name	Temp. (°C)	ABR					
		a	b	k	n	R ^2^	RMSE
Page	55	-	-	0.0037	1.7860	**0.9944**	**0.0254**
	85	-	-	0.0052	2.2050	**0.9972**	**0.0189**
	105	-	-	0.0004	3.2910	**0.9989**	**0.0136**
Henderson	55	1.2060	-	0.0550	-	0.9508	0.0753
	85	1.2550	-	0.1154	-	0.9168	0.1029
	105	1.3250	-	0.1236	-	0.8677	0.1463
Newton	55	-	-	0.0466	-	0.9196	0.0962
	85	-	-	0.0918	-	0.8568	0.1350
	105	-	-	0.0963	-	0.7982	0.1806
Logarithmic	55	1.3530	-0.2242	0.0354	-	0.9800	0.0480
	85	3.1520	-2.0300	0.0238	-	0.9855	0.0430
	105	1.9960	-0.7871	0.0487	-	0.9338	0.1035

SSE, sum of squared estimate of errors; RMSE, root mean square error; COV, coefficient of variation.

**Table 4.** Statistical data for isothermal drying of human faeces (HF).

Model Name	Temp. (°C)	HF					
		a	b	k	n	R ^2^	RMSE
Page	55	-	-	0.0109	1.4060	**0.9981**	**0.0134**
	85	-	-	0.0088	1.7760	**0.9977**	**0.0166**
	105	-	-	0.0013	2.7200	**0.9996**	**0.0075**
Henderson	55	1.1370	-	0.0471	-	0.9812	0.0421
	85	1.2310	-	0.0880	-	0.9590	0.0695
	105	1.2950	-	0.1128	-	0.8866	0.1285
Newton	55	-	-	0.0415	-	0.9624	0.0595
	85	-	-	0.0725	-	0.9158	0.0995
	105	-	-	0.0881	-	0.8178	0.1629
Logarithmic	55	1.2990	-0.2311	0.0302	-	0.9984	0.0122
	85	1.4530	-0.3076	0.0518	-	0.9873	0.0387
	105	2.8720	-1.7100	0.0269	-	0.9627	0.0738

SSE, sum of squared estimate of errors; RMSE, root mean square error; COV, coefficient of variation.

**Table 5.** Statistical data for isothermal drying of urine-diverting dry toilet (UDDT).

Model Name	Temp. (°C)	UDDT					
		a	b	k	n	R ^2^	RMSE
Page	55	-	-	0.0086	1.5300	**0.9988**	**0.0110**
	85	-	-	0.0054	2.0560	**0.9994**	**0.0088**
	105	-	-	0.0022	2.5470	**0.9995**	**0.0081**
Henderson	55	1.1670	-	0.0541	-	0.9740	0.0516
	85	1.2620	-	0.1012	-	0.9402	0.0876
	105	1.2930	-	0.1161	-	0.9032	0.1177
Newton	55	-	-	0.0468	-	0.9498	0.0717
	85	-	-	0.0817	-	0.8876	0.1201
	105	-	-	0.0364	-	0.9563	0.0417
Logarithmic	55	1.3050	-0.2093	0.0356	-	0.9942	0.0244
	85	1.5670	-0.3995	0.0543	-	0.9772	0.0541
	105	2.0260	-0.8503	0.0434	-	0.9628	0.0730

SSE, sum of squared estimate of errors; RMSE, root mean square error; COV, coefficient of variation.

**Table 6.** Statistical data for isothermal drying of ventilated improved pit (VIP).

Model Name	Temp. (°C)	VIP					
		a	b	k	n	R ^2^	RMSE
Page	55	-	-	0.0034	1.8090	**0.9915**	**0.0310**
	85	-	-	0.0013	2.6450	**0.9972**	**0.0202**
	105	-	-	0.0002	3.5500	**0.9976**	**0.0199**
Henderson	55	1.1940	-	0.0536	-	0.9392	0.0829
	85	1.2980	-	0.1080	-	0.8948	0.1249
	105	1.3250	-	0.1253	-	0.8441	0.1604
Newton	55	-	-	0.0451	-	0.9035	0.1044
	85	-	-	0.0854	-	0.8315	0.1580
	105	-	-	0.0964	-	0.7692	0.1951
Logarithmic	55	1.9730	-0.8955	0.0185	-	0.9916	0.0307
	85	1.9590	-0.7787	0.0422	-	0.9575	0.0794
	105	3.4670	-2.2810	0.0244	-	0.9342	0.1043

SSE, sum of squared estimate of errors; RMSE, root mean square error; COV, coefficient of variation.

Table 7 displays the effective moisture diffusivities deduced from the experimental data. The results show that *D _eff_* varied between 1.7∙10 ^–7^ and 10.0∙10 ^-7^ m ^2^/s at 55°C and 7.4∙10 ^-7^ and 11.0∙10 ^-7^ m ^2^/s at 105°C, for a sludge thickness of 3 mm, increasing with increased temperature and initial moisture content. These *D _ef_*_f_ values are higher compared to those reported for dewatered municipal sewage sludge, which are in the order of 0.17∙10 ^–8^ to 1.3∙10 ^–8^ m ^2^/s at drying temperatures of 65-135ºC and thickness of 2 - 10 mm ^36^. The increasing *D _eff_* values with drying temperatures was attributed to increasing water activity.

**Table 7.** Isothermal drying kinetic parameters of faecal sludges.

Temperature (°C)	Effective Moisture Diffusivity (m ^2^/s)			
	ABR	HF	UDDT	VIP
55	1.9∙10 ^-7^	1.7∙10 ^-7^	1.8∙10 ^-7^	1.9∙10 ^-7^
85	5.0∙10 ^-7^	3.7∙10 ^-7^	4.8∙10 ^-7^	5.6∙10 ^-7^
105	10.0∙10 ^-7^	7.4∙10 ^-7^	7.5∙10 ^-7^	11.0∙10 ^-7^
**Activation** **Energy (kJ/mol)**	**34 ± 0.8**	**30.5 ± 0.1**	**28.3 ± 1.3**	**36 ± 0.3**

ABR, anaerobic baffled reactor; HF, human faeces; UDDT, urine-diverting dry toilet; VIP, ventilated improved pit.

The results in Table 7 also show that *Ea* values were 34 ± 0.8 kJ/mol (ABR), 31 ± 0.1 kJ/mol (HF), 28 ±1.3 kJ/mol (UDDT) and 36 ± 0.3 kJ/mol (VIP). The Ea values for moisture diffusion in this study compared with those in Zhou and Jin ^36^, approximately 35 kJ/mol for dewatered municipal sewage sludge. Based on a review by Onwude et al ^35^, the majority (90%) of Ea values reported in food drying (that is, fruit and vegetables) are within 14 and 43 kJ/mol and higher values are in the range 78 and 130 kJ/mol. The relatively low values of the activation energy demonstrate that drying is a process kinetically controlled by physical phenomena, namely heat and mass transfer. A considerably higher activation energy would be expected for a process controlled by chemical phenomena.

The data obtained from this study can inform the design and development of FS drying. Using the expressions in Table 2 and the parameters in Table 3– Table 6, one can determine the drying rate and drying time for a given sample. This is illustrated in Table 8 at time, t = 10 min for drying rate and at MR = 0.5 for drying time. The data from the semi-theoretical model (Page) gave compatible results to those obtained from experimental results with RMSE <0.02 for drying rate and 1.30 for drying time. The results in Table 8 illustrates that a 55% increase in drying temperature (55 to 85ºC) reduced drying times by ~51% (ABR), ~39% (HF), ~40% (UDDT) and ~43% (VIP) but a 90% increase in temperature (55 to 105ºC) only increased drying times marginally (at most 49%) for all the sludges. This has implications for dryer operation: it shows that unnecessary heating can result during drying operation which can result in decreased efficiency and increased energy consumption, emissions and/or cost. Modelling the drying kinetics of FS is therefore important and can optimise their drying operation.

**Table 8.** Drying rate for t=20 min and drying time for MR=0.5 using Page Model at drying temperatures between 55 and 105ºC.

Drying Temperature (ºC)	dMR/dt (mg/min) at t=10 min (predicted)	dMR/dt (mg/min) at t=10 min (actual)	drying time (min) for MR=0.5 (actual)	drying time (min) for MR=0.5 (actual)
ABR				
55	-0.032	-0.029	18.73	19.27
85	-0.080	-0.070	9.20	9.28
105	-0.118	-0.112	9.64	9.62
HF				
55	-0.030	-0.029	19.17	19.95
85	-0.055	-0.056	11.69	11.28
105	-0.094	-0.090	10.06	9.68
UDDT				
55	-0.033	-0.031	17.62	17.70
85	-0.068	-0.068	10.60	10.37
105	-0.091	-0.090	9.57	9.43
VIP				
55	-0.032	-0.028	18.91	19.30
85	-0.086	-0.074	10.74	10.67
105	-0.124	-0.120	9.93	9.45
RMSE	0.02		1.30	

### Study limitation

Within the boundaries of experimental conditions, this study has demonstrated the influence of drying temperature on thin-layer drying of faecal sludges. Temperature is considered the most significant parameter affecting drying kinetics ^35, 37^, but more work is required to consider the effect of other material and/or process conditions including relative humidity, air velocities and sample thickness, that are not considered in this study. Due to the biological nature of samples that increases the risk of handling and storage in the laboratory and instrumental limitation (size of pan), the study has only considered limited sample number and size for each FS type but in typical onsite sanitation facilities, FS may vary in composition. To ensure uniform consistency of sample for analysis, FS was manually mixed in sample containers and mixing is expected to be part of pre-treatment operation for faecal sludges, particularly in toilets at risk of contamination e.g. VIP toilets. Pre-treatment options such as mixing, screening etc have not been considered and outside of the scope of this paper. Further work can expand on sample size, compositional variation and pre-treatment options.

For the kinetic analysis, the Page model was selected as most suited based on maximum R2 and minimum RMSE. It is worth mentioning that Page model like other semi-theoretical models with a lumped value of diffusivity does not consider temperature variation and assumes there is a uniform distribution of drying air within the sample. Furthermore, it assumes the following: homogenous size and properties of sludges under drying conditions; negligible shrinkages and pressure variation; uniform moisture and temperature distribution across the sample and during drying operations; constant apparent moisture diffusivity; internal heat transfer is dominated by conduction with negligible convective heat transfer; initial moisture content is independent of other parameters; evaporation occurs at the surface and equilibrium with drying air etc. These simplifications permit approximate analytical solutions but do not entirely describe complex drying operations and barriers to heat and mass transfer. For example, the model based on Fick’s law assumes constant apparent moisture diffusivity but in real conditions, there is increasing variation of D _eff_ which progressively becomes dominant at later drying regimes as moisture levels reduce to a minimum. This variation is caused by shrinkage, hardening, crust formation, cracking and other structural deformation effects. To minimise this, this study has considered Deff values for moisture content >20 wt. excluding warm-up/ramping conditions, but further work can expand on the shrinkage phenomenon in FS drying.

## Conclusions

Drying characteristics and kinetics of various faecal sludges were examined using thermogravimetric analysis under isothermal conditions. The results from this investigation suggested a high level of boundedness of the moisture in the FS samples, particularly for the HF and UDDT sludge, which would lead to a high energy consumption for drying. The absence of a constant rate period in this sample and UDDT suggests that the HF and UDDT sludges may contain limited unbound water that could be removed by dewatering. On the other hand, mechanical dewatering could be applied for the VIP and ABR FS to reduce the energy consumption for moisture removal. Drying could be improved by increasing the drying temperature, as this should favour the removal of bound moisture and enhance the mass transfer phenomena, leading to a fast process and a reduction in the drying time. The different drying behaviours of the samples suggest that the internal moisture transport phenomena occur differently as a function of the type of sludge. For all samples, the values of the activation energy ranged from 28–36 kJ/mol, which reflects a process controlled by transfer phenomena, and the effective diffusivity was in the order 10 ^-7^ m ^2^/s, which was higher than values from sewage sludge drying. The Page model described the drying kinetics with the best fit, so this type of model could be used for the design and operation of faecal sludge drying systems.

## Data availability

### Underlying data

Figshare: DATA-DRYING-ISOTHERM-FAECAL-SLUDGES.xlsx. https://doi.org/10.6084/m9.figshare.12349622.v1 ^29^.

Data are available under the terms of the Creative Commons Zero “No rights reserved” data waiver (CC0 1.0 Public domain dedication).

## Disclaimer

The work was completed at Cranfield University and findings and conclusions contained within are those of the authors and do not necessarily reflect positions or policies of the Bill and Melinda Gates Foundation.
