# Supplementary material for: Isothermal drying characteristics and kinetics of human faecal sludges
Source: Gates Open Res. 2021 Apr 21;4:67. Originally published 2020 Jun 25. [Version 2] doi: 10.12688/gatesopenres.13137.2 (PMC8135097; doi:10.12688/gatesopenres.13137.2)
Supplement: Peer review history

# Peer review: Reviewer response for version 2

**DOI:** 10.21956/gatesopenres.14477.r30605

**Published:** 2021-05-19

**Authors:** Yu-Ling Cheng

**Affiliations:** 1 Centre for Global Engineering and the Department of Chemical Engineering and Applied Chemistry, University of Toronto, Toronto, ON, Canada

I approved for indexing with no further comments.

Is the work clearly and accurately presented and does it cite the current literature?

Partly

If applicable, is the statistical analysis and its interpretation appropriate?

Partly

Are all the source data underlying the results available to ensure full reproducibility?

No source data required

Is the study design appropriate and is the work technically sound?

Partly

Are the conclusions drawn adequately supported by the results?

Partly

Are sufficient details of methods and analysis provided to allow replication by others?

Partly

Reviewer Expertise:

NA

I confirm that I have read this submission and believe that I have an appropriate level of expertise to confirm that it is of an acceptable scientific standard.

# Peer review: Reviewer response for version 1

**DOI:** 10.21956/gatesopenres.14325.r29598

**Published:** 2020-10-12

**Authors:** Suyun Xu

**Affiliations:** 1 School of Environment and Architecture, University of Shanghai for Science and Technology, Jungong Road, Shanghai, 200093, China

This study investigated the drying characteristics and kinetics of various faecal wastes using thermogravimetric analysis and isothermal heating conditions to provide information for the thermal treatment of faecal sludge.

 This topic is important for the treatment and resource utilization of faecal sludge. Generally, this manuscript is well prepared, The motivation, methods, results and analysis are, for the most part, clearly presented and easy to understand.

 I have several comments for the transferability of this study results into practical operation below:

1. As all experiments were carried out with TGA, and the amount of sample used for analysis is limited. How to ensure the accuracy of these data obtained from the low-dose sample?
2. How to use these kinetics data and isothermal heating parameters for the design of drying equipment for FS, VIP and UDDT? A bit more discussion can be added before the Conclusion.
3. All experiments were carried out using an airflow rate of 40 mL/min. In fact, the airflow rate is one of the key factors influencing the drying rate. However, this parameter was not considered in this study. How about the applicability of Page Model?

Is the work clearly and accurately presented and does it cite the current literature?

Yes

If applicable, is the statistical analysis and its interpretation appropriate?

Not applicable

Are all the source data underlying the results available to ensure full reproducibility?

Partly

Is the study design appropriate and is the work technically sound?

Yes

Are the conclusions drawn adequately supported by the results?

Yes

Are sufficient details of methods and analysis provided to allow replication by others?

Yes

Reviewer Expertise:

Biowaste treatment

I confirm that I have read this submission and believe that I have an appropriate level of expertise to confirm that it is of an acceptable scientific standard.

# Peer review: Reviewer response for version 1

**DOI:** 10.21956/gatesopenres.14325.r28987

**Published:** 2020-08-13

**Authors:** Yu-Ling Cheng

**Affiliations:** 1 Centre for Global Engineering and the Department of Chemical Engineering and Applied Chemistry, University of Toronto, Toronto, ON, Canada

**General comments:**

 In this paper, samples of faecal sludge/human faeces were obtained from four sources: anaerobic baffled reactor, VIP latrines, an ablution block, and direct donations from volunteers. The drying kinetics of test masses from collected FS samples at varying temperatures were obtained using TGA, and the results were analyzed by fitting to four mathematical expressions. Then a mechanistic model based on Fickian diffusion was used to extract values for effective diffusion coefficients for each faecal sludge sample and at each temperature, and a value for activation energy was then obtained.

 The topic of this manuscript is important. Motivation, methods, results and analysis are, for the most part, clearly presented and easy to understand. The authors are to be commended for their experimental work as well as the effort to extract mechanistic information from the data. I have a number of significant comments and questions that I have attempted to organize into logical categories below. I hope my comments will contribute to an improved paper and make this important work more useful to the community. Note that my comments are based on the paper, I did not examine the primary data.

**A Transferability of results and conclusions to drying operations:**

 As the authors point out, drying is important in faecal sludge transport and processing and was the motivation for the study. It would help the reader relate the results from this study to potential drying operations if the authors can clarify the following:

1. How many latrines/toilets/ablution blocks were the faecal sludge samples collected from? For example, are the reported VIP latrine data from samples collected a single time from a single latrine? Or multiple times from the same latrine? Similarly, are the human faeces samples from a single donor, or was it a sample from agglomerated donations from multiple volunteers? Whatever the answers are, can the authors comment on how representative their samples are of each broad category of faecal sludge?
2. Were the collected samples homogeneous? If not, were they homogenized before the 40 mg TGA test masses were taken? If so, how was this done?
3. How do the sample preparation methods between the time of collection and TGA kinetics analysis compare to how faecal sludge would be prepared in drying operations? Methods of preparation can affect the nature of the matrix or medium through which water diffuses.
4. Can the authors compare typical faecal sludge thickness in drying operations vs TGA sample thickness? Thickness is an important parameter in drying kinetics.

**B TGA analysis and results:**

Air flow rate of 40 mL/min was used for TGA drying experiments. Was the convective mass transfer resistance under these conditions negligible compared to internal diffusive resistance?
What was the relative humidity in the atmosphere for the drying experiments? This is useful to know because it gives the sink condition for drying.
The equilibrium moisture content was assumed to be zero in equation 1. The equilibrium moisture content should be a function of the temperature and relative humidity, but at low RH can be a small enough number that it can be assumed to be zero. A comparison of the TGA drying data to the measured initial moisture content should be presented to indicate whether this is a good assumption.
Each test was conducted at least in duplicate. Does this mean at least two 40 mg test masses were removed from different parts of each faecal sludge sample and run at the specified temperature? Or that there were multiple collected faecal sludge samples? (Same question as A1.) How much variation was there in the results among replicate masses? Can standard deviations be reported on extracted quantities such as diffusion coefficient and activation energy?
Table 1. The moisture content for HF and UDDT are significantly lower than in healthy faeces. Presumably, the UDDT samples dry in the pit before collection. What is the explanation for the low moisture content (62%) of human faeces collected without further storage or processing?
Moisture in the manuscript is reported in units of %, but not defined. Is it mass of water divided by total mass of faecal sludge, ie the mass basis concentration of water in the faecal sludge matrix?
The x-axis in Figure 2b should be labelled as moisture content, not time.
8. In Figure 1b, the drying rate axis is labelled as %. In Figures 2b, 3b , 4b and 5b. the drying rate axes are labelled as -dMR/dT, with numbers between 0 and 1 on the axis but no units.
  (a) I believe the y-axis labels in Figures 2b to 5b are drying rates with units of mg/min. Please confirm and label with the proper units.
  (b) What does the % in the y-axis in Figure 1b represent? And why is it presented differently than Figures 2b, 3b, 4b and 5b? A time component is needed for drying rate.
  (c) To be consistent with the use of t for time, and T for temperature in other parts of the manuscript (eg equations 2 to 6), the drying rate symbol on the y-axis should be dMR/dt.

**C Models and data analysis**

 The following comments relate to data analysis using the four fitting models and the Fickian diffusion model:

1. Small inconsistency: equation 7 gives the definition of X ^2^ for goodness of fit, but statistical analysis shown in Tables 3 to 6 used other statistical quantities SSE, R2, and RMSE to conclude that the Page model gave the best fit for most conditions. The short section on statistical analysis of drying models needs to be edited to reflect what was actually done.
2. The coefficient of variation numbers in Tables 3 to 5 are mistakenly listed. The numbers listed for COV are 1, 2 or 3, corresponding to the number of fitting parameters in each of the models.
3. My analysis of the four models and the fitting results - as outlined below - raises questions about the way the statistical analysis was done that the authors should address. Even though the models are only loosely mechanistically based, the parameters obtained from fitting should still make sense, but the results of the logarithmic model and the Page model are inconsistent with inferred mechanisms.
  (a) The Newton model MR(t) = exp (-kt) is the same as the Henderson model MR(t) =a exp (-kt) but with the pre-exponential factor forced to take on a value of one. Since MR(t =0) = 1, it makes some sense to choose the Newton model. The fit of the Newton model is not quite as good as the Henderson Model, but that is to be expected since there is only one fitting parameter. Also, note that the fitted values for ”a” in the Henderson model are all close to 1 - ranging from 1:07 to 1:016; so the two models give very similar results.
  The form of these two models is consistent with the simplified Fickian diffusion model (equation 4) which gives a pre-exponential factor of 8/π ^2^ = 0:81 rather than 1 due to the truncation of all but the first term of the infinite series. The good fit of the Henderson and Newton models suggest that Fickian diffusion is a key determinant of drying kinetics.
  (b) The logarithmic model MR(t) = a + b exp (-kt) is similar to the Henderson model but with a component of water that cannot be removed: the parameter ”a” in the logarithmic model would represent an irreversibly bound amount of water, while the drying of the removable fraction is consistent with Fickian diffusion. Given this physical interpretation, the negative values of ”a” obtained from fitting, ranging from -0:04 to -1:09, is perplexing and suggests that either something has been reported in error or that the statistical analysis was done incorrectly. Clarification is required.
  (c) The Page model MR(t) = exp (-kt ^n^) is unique among the four fitting models due to the t ^n^ term. The Fickian diffusion model assumes that D _eff _is constant throughout the drying process, but the reality is that the matrix through which water must be transported changes as the sludge dries, and Deff should decrease with time. Having a t ^n^ terms provides a way to account for such time dependent changes in the intrinsic nature of the medium, and makes the Page model attractive. However, since we expect D _eff_ to decrease with time, the exponent n should be less than 1. So it is perplexing that the best fit values of n shown in Tables 3 to 6 are all greater than 1 - ranging from 1:29 to 1:60. Some clarification or re-checking of the statistical analysis should be done.
  (d) Given the questions raised above about the logarithmic model and the Page model, the conclusion that the Page model gives the best fit should be reviewed.
  (e) In addition, given the relatively small number of samples, are the relatively small differences in goodness of fit of the models statistically significant?
4. The authors use the simplified result of the Fickian model to extract Deff from the data, and then to compute an activation energy. There is some internal inconsistency since they had concluded the Page model to be the best fit. Nevertheless, the fitting results of the Henderson/Newton models make sense and can justify interpretation using the Fickian model.
  A big assumption in Fick’s model is that the effective diffusion coefficient is a constant throughout the drying process. This assumption fails as the water concentration changes over a wide range of values - eg MR ranging from 1:0 down to 0 means the water concentration changes from 92% to zero for faecal sludge from VIP toilets, and from 48% to zero for UDDT latrines. The medium in which water diffuses would change significantly over the course of each drying kinetics experiment, and therefore Deff would change significantly - most likely decreasing over time. It would be useful to see the ln (MR) vs ln (t) plots to see how good the fit is to a straight line. I would anticipate deviations from linearity over time.
  Another possible reason the Fickian model would deviate from ideality over time is that depending on the relative kinetics of internal diffusion and evaporation at the surface, a skin can form at the surface of the matrix. The skin would act as a barrier membrane and slow down evaporation in the latter part of the drying experiment. This is a commonly observed phenomenon in the drying of foods and would also lead to a decrease in apparent Deff over time. Note that skin formation would be expected to impact drying kinetics more significantly in thicker sludge - such as would be expected in drying operations.
5. It would be useful to show the diffusivities and activation energies in Table 7 with confidence intervals so the reader can see if the differences for different sludges are statistically significant.
6. Assuming the differences in diffusivities are statistically significant, can the authors explain some of the observed differences? For example, why is the diffusivity in UDDT at several of the temperatures higher than the other sludges even though it is has the lowest initial water content?
7. A point of precision: I think of diffusion as the phenomenon of a chemical species moving in a medium due to molecular level motion. So it is water as the chemical species - not moisture – that diffuses in faecal sludge. Accordingly, D _eff _is the effective diffusion coefficient of water, not of moisture.

**D Interpretation and conclusions:**

 The authors attribute a great deal of significance to bound water vs free water, and present interpretations of varying regions of drying curves to the critical moisture content. I agree that there it is important to distinguish between the two types of water which helps to understand when mechanical dewatering processes can be used vs when heat addition to facilitate evaporation/drying is necessary. There are two additional points to make:

1. The presence and extent of bound water can be more easily determined by more direct methods - particularly measurements of the thermodynamic activity of water in the sludge at varying stages of drying, and measurements of boiling points. Bound water would have a slightly higher boiling point than free water that would be observable by differential scanning calorimetry. Using drying kinetics curves to infer bound vs free water is indirect, and other phenomena can complicate data interpretation.
2. In broad terms, transport rates are related to the driving force divided by resistance. The free water/bound water argument addresses only the driving force component of transport rate - when all the free water has been removed, the thermodynamic activity falls below one and the driving force decreases. However, resistance is also increasing as the matrix becomes dryer or a skin is formed and can also impact drying kinetics.

Is the work clearly and accurately presented and does it cite the current literature?

Partly

If applicable, is the statistical analysis and its interpretation appropriate?

Partly

Are all the source data underlying the results available to ensure full reproducibility?

No source data required

Is the study design appropriate and is the work technically sound?

Partly

Are the conclusions drawn adequately supported by the results?

Partly

Are sufficient details of methods and analysis provided to allow replication by others?

Partly

Reviewer Expertise:

NA

I confirm that I have read this submission and believe that I have an appropriate level of expertise to confirm that it is of an acceptable scientific standard, however I have significant reservations, as outlined above.
